# Deletion of VDAC1 Hinders Recovery of Mitochondrial and Renal Functions After Acute Kidney Injury

**DOI:** 10.3390/biom10040585

**Published:** 2020-04-10

**Authors:** Grazyna Nowak, Judit Megyesi, William J. Craigen

**Affiliations:** 1 Department of Pharmaceutical Sciences, College of Pharmacy, University of Arkansas for Medical Sciences, Little Rock, AR 72205, USA; 2 Department of Internal Medicine, Division of Nephrology, College of Medicine, University of Arkansas for Medical Sciences, Little Rock, AR 72205, USA; megyesijuditk@uams.edu; 3 Department of Molecular and Human Genetics, Bailor College of Medicine, Houston, TX 77030, USA; wcraigen@bcm.edu

**Keywords:** voltage-dependent anion channel-1 (VDAC1), ischemia and reperfusion, mitochondrial fission, mitochondrial dysfunction, electron transport chain, respiratory complexes, ATP synthase, ATP content

## Abstract

Voltage-dependent anion channels (VDACs) constitute major transporters mediating bidirectional movement of solutes between cytoplasm and mitochondria. We aimed to determine if VDAC1 plays a role in recovery of mitochondrial and kidney functions after ischemia-induced acute kidney injury (AKI). Kidney function decreased after ischemia and recovered in wild-type (WT), but not in VDAC1-deficient mice. Mitochondrial maximum respiration, activities of respiratory complexes and F_o_F_1_-ATPase, and ATP content in renal cortex decreased after ischemia and recovered in WT mice. VDAC1 deletion reduced respiration and ATP content in non-injured kidneys. Further, VDAC1 deletion blocked return of activities of respiratory complexes and F_o_F_1_-ATPase, and recovery of respiration and ATP content after ischemia. Deletion of VDAC1 exacerbated ischemia-induced mitochondrial fission, but did not aggravate morphological damage to proximal tubules after ischemia. However, VDAC1 deficiency impaired recovery of kidney morphology and increased renal interstitial collagen accumulation. Thus, our data show a novel role for VDAC1 in regulating renal mitochondrial dynamics and recovery of mitochondrial function and ATP levels after AKI. We conclude that the presence of VDAC1 (1) stimulates capacity of renal mitochondria for respiration and ATP production, (2) reduces mitochondrial fission, (3) promotes recovery of mitochondrial function and dynamics, renal morphology, and kidney functions, and (4) increases survival after AKI.

## 1. Introduction

Acute kidney injury (AKI) is a clinical syndrome characterized by a rapid decline in kidney function and failure to regulate fluid, electrolyte, and acid–base balance. AKI originates from a variety of causes, including renal hypoperfusion (due to low cardiac output or reduced renal perfusion pressure), obstruction of the urinary tract, infections and sepsis, and exposure to nephrotoxins [1,2]. A common mechanism for AKI is ischemia (inadequate O_2_ and nutrient delivery)-reperfusion injury, which involves mitochondrial dysfunction, oxidative stress, insufficient production of ATP, renal cell apoptosis and necrosis, and inflammation [1,2,3]. Mitochondrial dysfunction and ATP deficits are the most pronounced in tubular cells of renal cortex and precede the clinical manifestations of AKI [2,3,4,5]. As our understanding of the complex mechanisms involved in mitochondrial dysfunction remains inadequate, the therapies that reduce mitochondrial damage and/or stimulate mitochondrial biogenesis in the injured kidney are very limited.

An efficient exchange of solutes and nucleotides between mitochondria and the cytoplasm is required to maintain cellular energy metabolism. Among the three identified subtypes of the voltage dependent anion channel (VDAC1, VDAC2, and VDAC3), VDAC1 is most widely expressed and present in the majority of cell types [6,7,8,9,10,11]. VDAC1 is the major gateway for solute transport between the cytoplasm and mitochondria. VDAC1 is the most abundant integral protein component of the outer mitochondrial membrane and, in physiological conditions, mediates the bidirectional transport of ions, nucleotides (e.g., NAD^+^/NADH, ADP/ATP), and solutes smaller than 5 kDa in size [6]. Thus, VDAC1 is a crucial regulator of mitochondrial energy metabolism and Ca^2+^ homeostasis [7,8,9]. However, under pathophysiologic conditions, VDAC1 is involved in apoptosis; both at the early stages (Ca^2+^ influx into mitochondria) as well as in later stages, when pro-apoptotic proteins are released from the mitochondria, and in ferroptosis [8,12,13,14,15]. Apoptosis is associated with increased expression of VDAC1 in the outer mitochondrial membrane and oligomerization of VDAC1 monomers to form large channels that allow for release of mitochondrial pro-apoptotic proteins into the cytoplasm where they mediate additional apoptotic events [10,15,16,17]. Regulation of cell survival signals by VDAC1 occurs through its interactions with proteins of the Bcl-2 family, hexokinase, protein kinases, cytoskeletal proteins (tubulin, α-synuclein, plectin, and desmin), and mitochondrial lipids (cardiolipin and phoshatidylethanolamine) [16,17,18,19,20,21,22,23,24].

VDAC1 closure results in the decrease or loss of the outer mitochondrial membrane permeability and accumulation of metabolites, nucleotides, and protons in the intermembrane space. If this condition persists, the outer membrane integrity is lost, mitochondrial homeostasis is disrupted, and cytochrome c diffuses to the cytosol thereby initiating apoptosis [25]. In contrast, VDAC1 opening prevents events leading to apoptosis [19,26]. Thus, VDAC1 permeability has a critical impact not only on mitochondrial homeostasis, metabolic functions, and stress, but also on cellular ATP levels, injury, and fate.

In contrast to a substantial amount of knowledge of the role of VDAC1 in physiology and pathology of neuronal, cardiac, and cancer cells, very little is known about the role of VDAC1 in kidney physiology, injury, and regeneration. GSK3β-mediated phosphorylation of a threonine residue(s) of VDAC1 leads to mitochondrial permeability transition and death in cultured renal epithelial tubular cells [27]. Inhibition of GSK3β blocks phosphorylation of VDAC1, attenuates mitochondrial permeability transition, reduces mitochondrial dysfunction and oxidative stress, and ameliorates oxidant-induced AKI [27]. Deletion of VDAC3 increases mitochondrial production of the reactive oxygen species, alters renal sodium transport, and leads to hypertension whereas deletion of VDAC2 is lethal during embryonic development [28,29]. Oligomerization of VDAC1 has been implicated in cisplatin-induced apoptosis and nephrotoxicity due to a formation of pores in the outer mitochondrial membrane that allow for the escape of cytochrome c from the mitochondrial intermembrane space to the cytosol [30]. As VDAC1 conductance can differentially mitigate or exacerbate pathological conditions in different organs and cell types, the purpose of this study was to determine the role of VDAC1 in (1) ischemia-induced mitochondrial dysfunction and injury to the kidney, and (2) kidney repair after ischemia.

## 2. Materials and Methods

Many of the methods used in this study have been described in detail in our previously published papers. Publications describing these methods in detail are cited in the respective paragraphs.

### 2.1. VDAC1-Deficient Mice

VDAC1^−^/^−^ mice were generated by the gene targeting approach in embryonic stem cells as described previously [31]. The targeting vector had a deleted 3.7-kb HindIII fragment containing exons 2–5 of the VDAC1 gene. While heterozygous VDAC1^+^/^−^ mice have no obvious phenotype and are fertile, mating of heterozygous parents results in a decreased number of homozygous VDAC1^−^/^−^ offspring (VDAC1 deficient, VDAC1 KO) in comparison with the expected 1:2:1 Mendelian ratio, which suggests partial embryonic lethality [31]. Surviving homozygous VDAC1^−^/^−^ offspring display no obvious phenotype, are not different from wild-type littermates physically and behaviorally [31].

### 2.2. Animal Breeding and Genotyping

All animal procedures were carried out in accordance with the Public Health Service and NIH Policy on Humane Care and Use of Laboratory Animals and ethically reviewed by the Institutional Animal Care and Use Committee (IACUC) at the University of Arkansas for Medical Sciences. All experiments described in this manuscript (including breeding and genotyping) were approved by the IACUC protocol #3818. The mice were housed in a barrier environment at constant room temperature 20 °C, 12 h light/dark cycle with a free access to standard diet and water. Heterozygous VDAC1^+^/^−^ mice (CD1 strain) were generously provided by Dr. William J. Craigen (Baylor College of Medicine, Houston, TX, USA). Heterozygous parents were bred to obtain homozygous VDAC1-deficient (VDAC1 KO) and wild-type (WT) littermates. Genotyping of the offspring was carried out as previously described [31] with minor modifications [32]. Tail snips of 3-week old offspring were lysed overnight at 55 °C in a digestion media containing 0.2 mL DirectPCR Lysis Reagent (Viagen Biotech; Los Angeles, CA, USA) and 12 μg Proteinase K (InVitrogen; Carlsbad, CA, USA) and the resulting lysates containing tail DNA were analyzed by a four-primer multiplex polymerase chain reaction (PCR) assay using primer sets supplied by Integrated DNA Technologies (Coralville, IA, USA) and PCR conditions (94 °C for 1 min, 56 °C for 1 min, and 72 °C for 1 min and 30 s, 40 cycles) described previously [31]. VDAC1-deficient mice were selected from the offspring based on the results of PCR. The VDAC1-deficient mice developed normally, reached the same size, body weight and life span, and were fertile. No changes in kidney size and morphology between WT and VDAC1 KO mice were observed.

### 2.3. Ischemia/Reperfusion Injury

Due to the presence of higher amounts of testosterone, male mice are more susceptible to kidney ischemia/reperfusion injury when compared with females [33,34]. Therefore, male mice were used in this study. The procedure was described in detail previously [32,35]. Briefly, 3–4 month old mice were anesthetized using sodium pentobarbital (50 mg/kg b.w.; Ovation Pharmaceuticals, Deerfield, IL, USA), a midline incision of the abdomen was performed under sterile conditions, and kidneys were exposed and decapsulated. Both renal hila were clamped for 35 min using small vascular clamps and mice were placed onto a heating pad that maintained a temperature of 37 °C. During this period, mice received approximately 0.5 mL sterile saline intraperitoneally. After removing the clamps, reperfusion of both kidneys was confirmed visually and the abdomen was closed with silk suture [32,35]. Sham operations involved kidney manipulations but no ischemia. After the surgery, the mice were returned to their cages and kept at the same housing conditions as the sham controls with free access to food and water. At 24 and 168 h after reperfusion, mice were sacrificed and blood and kidneys were harvested to determine renal functions and tissue morphology, and to isolate mitochondria from renal cortex.

### 2.4. Morphological Assessment of Kidney Tissue

Harvested kidneys were immediately immersed and fixed in 4% neutral-buffered formaldehyde. The tissues were embedded in paraffin, thin (4–8 μm) sections were cut, processed for light microscopy, and stained with hematoxylin-eosin and periodic acid Schiff (PAS) as described previously [32,35]. The morphological damage to the kidney was assessed using light microscopy and the histologic criteria that included tubular necrosis, brush border loss, tubular dilatation, tubular cast formation, distal nephron damage, degeneration, red blood cell extravasation, and inflammation. Degeneration of the proximal tubules did not score necrotic tubules, but involved cell swelling and the formation of vacuoles and PAS positive granules in the cytoplasm in response to the damage of the membranes, disruption of ion transport, and the accumulation of intracellular lipids and proteins or lipoproteins. Regeneration was scored in the tubules that lost cells by necrosis but were relined by new undifferentiated epithelial cells. The score was given based on the extent of these changes and the state of differentiation of the epithelium. Inflammation was determined by the density and the number of inflammatory cells throughout the kidney sections. These parameters were scored on a scale of 0 to 4: not present (0), mild (1), moderate (2), severe (3), and very severe (4) in three different animals as described previously [32,35]. The images were captured using a Nikon Eclipse E800 microscope (Nikon Instruments Inc., Melville, NY, USA) and a Nikon 10× Plan Apo objective.

### 2.5. Assessment of Interstitial Changes in Kidney Tissue

Kidneys from wild type and VDAC1-deficient mice were harvested 7 days post reperfusion. Formalin-fixed and paraffin-embedded 4–8 μm tissue sections from sham and ischemic kidneys were stained with picro-sirius red for 1 h as previously described [36]. Collagen staining in the renal interstitium was assessed by light microscopy using circularly polarized light and Nikon Eclipse E800 microscope (Nikon Instruments Inc., Melville, NY, USA) and a Nikon 10× Plan Apo objective.

### 2.6. Plasma Creatinine Levels

Creatinine levels in blood plasma were assessed spectrophotometrically using Creatinine Reagent Set (Biotron Diagnostics; Hemet, CA, USA) and the manufacturer’s protocol as described previously [32].

### 2.7. Isolation of Mitochondria

Mice were sacrificed at 24 and 168 h after ischemia, and renal cortices were dissected and homogenized in the ice-cold isolation buffer (10 mM Hepes, 225 mM mannitol, 75 mM sucrose, 2 mM EGTA, and 0.1% BSA, fatty acid free, pH 7.4) as described previously in detail [32]. The homogenate was centrifuged at 1000× *g* for 5 min at 4 °C and the resulting supernatant was spun down at 15,000× *g* for 15 min at 4 °C. The mitochondrial pellet was washed twice using the isolation buffer and centrifuged again at 15,000× *g* for 10 min at 4 °C. The final mitochondrial pellet resuspended in the isolation buffer was used to measure state 3 respiration. For measurements of activities of respiratory complexes and F_0_F_1_-ATPase, the final mitochondrial pellet was resuspended in the assay buffer (10 mM KH_2_PO_4_, 5 mM MgCl_2_, pH 7.2).

### 2.8. State 3 Respiration

State 3 respiration was determined as described before [32] by measuring oxygen consumption in freshly isolated mitochondria suspended in an assay buffer containing 20 mM Hepes (pH 7.4), 137 mM KCl, 2 mM KH_2_PO_4_, 0.5 mM EGTA, 5 mM MgCl_2_. Complex I-coupled respiration was determined using glutamate (5 mM) and malate (5 mM) as oxidative substrates that donate majority of electrons to complex I. Complex II-coupled respiration was assessed using 10 mM succinate (in the presence of 0.1 μM rotenone) as an oxidative substrate that donates majority of electrons to complex II (complex II-coupled respiration). To determine complex IV-coupled respiration, 1 mM ascorbate (in the presence of 1 mM N,N,N,N-tetramethyl-p-phenylenediamine; TMPD) was used as the oxidative substrate. Following the addition of substrates to the assay buffer, state 3 respiration was initiated by adding ADP (0.4 mM). State 4 respiration was measured in the presence of oligomycin (2.5 μg/mL; Calbiochem, San Diego, CA, USA).

### 2.9. Activities of Respiratory Complexes

Activities of complexes of the electron transport chain were measured in isolated mitochondria as described previously in detail [32,37].

#### 2.9.1. Complex I (NADH:Ubiquinone Oxidoreductase)

Complex I activity was determined spectrophotometrically at 30 °C by measuring the oxidation of NADH (0.25 mM) at 340 nm in the assay buffer (10 mM KH_2_PO_4_, 5 mM MgCl_2_, 0.25% BSA; pH 7.2) containing 62.5 μM ubiquinone, antimycin A (2 μg/mL), and mitochondria. The decrease in absorbance due to NADH oxidation was recorded for 3 min, rotenone (10 μg/mL) was added, and the absorbance was recorded for another 2 min. Complex I activity was calculated as the rotenone-sensitive NADH:Ubiquinone Oxidoreductase activity.

#### 2.9.2. Complex II (Succinate:Ubiquinone Oxidoreductase)

Complex II activity was determined at 30 °C by following the reduction of dichlorophenolindophenol (0.25 mM) at 590 nm in the presence of 20 mM succinate, antimycin A (2 μg/mL), rotenone (10 μg/mL), 0.25% BSA, and ubiquinone (62.5 μM).

#### 2.9.3. Complex III (Ubiquinol:Cytochrome c Oxidoreductase)

Complex III activity was assessed at 30 °C, by measuring the reduction of cytochrome c (60 μM) at 550 nm in the assay buffer containing decylubiquinol (50 μM), 0.25% BSA, rotenone (10 μg/mL), and KCN (0.24 mM). The increase in absorbance caused by the reduction of cytochrome c was recorded in the absence and presence of antimycin A (2 μg/mL). Activity of complex III was calculated as the antimycin A-sensitive activity.

### 2.10. F_0_F_1_-ATPase Activity

ATPase activity of ATP synthase was assessed in freshly isolated mitochondria by measuring hydrolysis of ATP according to Law et al. as described in detail in our previous publications [37,38,39]. Samples were run in the absence and presence of oligomycin (10 μg/mL) and the oligomycin-sensitive ATPase activity was calculated.

### 2.11. Tissue ATP Content

Renal cortical tissue slices were dissected immediately after animal sacrifice, placed into a solution of ice cold 3% perchloric acid, and snap-frozen in liquid nitrogen and stored at −80 °C. On the day of analysis, frozen samples were thawed, sonicated on ice, and spun down at 10,000× *g* for 1 min at 4 °C. The supernatant was collected, placed on ice, neutralized to pH of 7.4 with KOH, and used to determine the content of ATP. ATP concentration in neutralized supernatants was measured by the luciferase luminescence method using Bioluminescence Assay Kit HS II (Roche, Mannheim, Germany) and the manufacturer’s protocol as described previously [39]. The pellets containing denatured tissue proteins were solubilized in 100 mM Tris-HCl buffer containing 150 mM NaCl buffer and 0.05% Triton X-100 and sonicated on ice and used to determine protein concentration in samples. ATP content was expressed as nmoles of ATP per mg of tissue protein.

### 2.12. Immunoblotting

Immunoblot analysis was used to assess protein levels of VDAC isoforms, subunits of complexes I and III, F_0_F_1_-ATPase, and the dynamin-related protein 1 (DRP1) in renal cortical mitochondria as described in detail in previously [40,41]. Anti-VDAC1, anti-VDAC2, and anti-VDAC3 antibodies were purchased from EMD Millipore (Danvers, MA, USA), Abcam (Cambridge, MA, USA), and Santa Cruz Biotechnologies (Santa Cruz, CA, USA), respectively. Antibodies against subunits of complex I were purchased from Molecular Probes/ThermoFisher Scientific (Waltham, MA, USA). Antibodies against α- and γ-subunits of F_0_F_1_-ATPase and the core protein 1, core protein 2, and Rieske subunits of complex III were supplied by Abcam. Antibodies against β-subunit of F_0_F_1_-ATPase were supplied by Life Technologies Corporation (Carlsbad, CA, USA). Protein levels of citrate synthase were used as the loading control. Anti-citrate synthase antibodies were provided by Cell Signaling Technology (Danvers, MA, USA).

### 2.13. Protein Content

All results were normalized to sample protein content, which was measured by the bicinchoninic acid assay using BSA as the standard as described previously [32,40,41].

### 2.14. Statistical Analysis

Data are presented as means ± S.E. and were analyzed for significance by the two-sided Student’s *t*-test for independent samples or by ANOVA. Multiple means were compared using Fisher’s protected least significance difference (LSD) test with a level of significance of *p* < 0.05.

## 3. Results

### 3.1. Ischemia Decreases the Levels of VDAC1 in Renal Cortical Mitochondria

**Figure 1:**
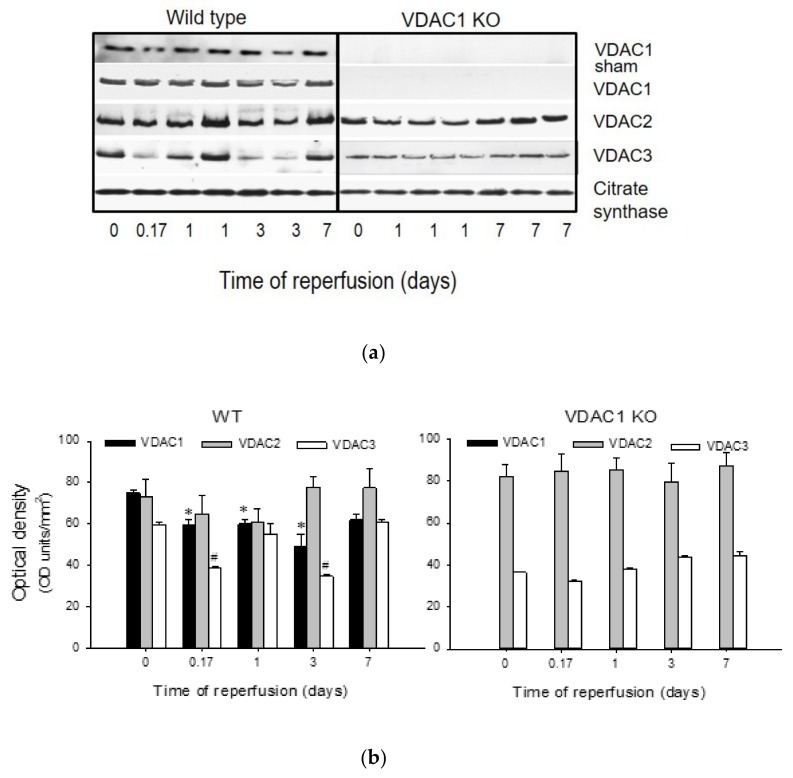
Ischemia induces changes in protein levels of voltage-dependent anion channel (VDAC) isoforms in renal cortical mitochondria. (**a**) Protein levels of in renal cortical mitochondria were assessed at different time points after bilateral renal ischemia (35 min) in wild type (left panel) and VDAC1-deficient (right panel) mice. Mitochondria were isolated from renal cortices of kidneys harvested at 4 h and days 1, 3, and 7 (wild-type—WT) or days 1 and 7 (VDAC1 KO) after ischemia and processed for immunoblotting. A total of 20 μg of protein from each sample were used for immunoblotting. The blots shown in this figure are representative of three independent experiments. (**b**) Densitometric analysis of immunoblots of VDAC1, VDAC2, and VDAC3 in WT (left panel) and VDAC1 KO (right panel) mitochondria. *, # Values significantly different (*p* < 0.05) from respective controls (time 0).

Protein levels of VDAC1, VDAC2, and VDAC3 were assessed in mitochondria isolated from renal cortex of sham-operated and ischemic WT and VDAC1 KO mice at different times post reperfusion. Mitochondrial VDAC1 levels did not change in WT sham animals, but decreased after ischemia with the lowest level found on day 3 post reperfusion. On day 7 after ischemia, VDAC1 levels were not different from sham controls (Figure 1). Likewise, mitochondrial levels of VDAC2 decreased after ischemia and returned to the control levels by day 7 (Figure 1). Mitochondrial levels of VDAC3 decreased shortly (4 h) after ischemia, remained decreased until day 3 post reperfusion, and recovered by day 7 (Figure 1). As shown in Figure 1, VDAC1 protein was absent in mitochondria from VDAC1 KO mice. VDAC1 deletion did not alter the changes in renal mitochondrial levels of VDAC2 and VDAC3 in response to ischemia (Figure 1).

These data show that ischemia decreases the levels of all known VDAC isoforms and that the kidney recovery after ischemic injury is associated with the return of mitochondrial levels of VDAC isoforms in the kidneys of wild type mice.

### 3.2. Deficiency of VDAC1 Delays Recovery of Renal Function After Ischemia

**Figure 2:**
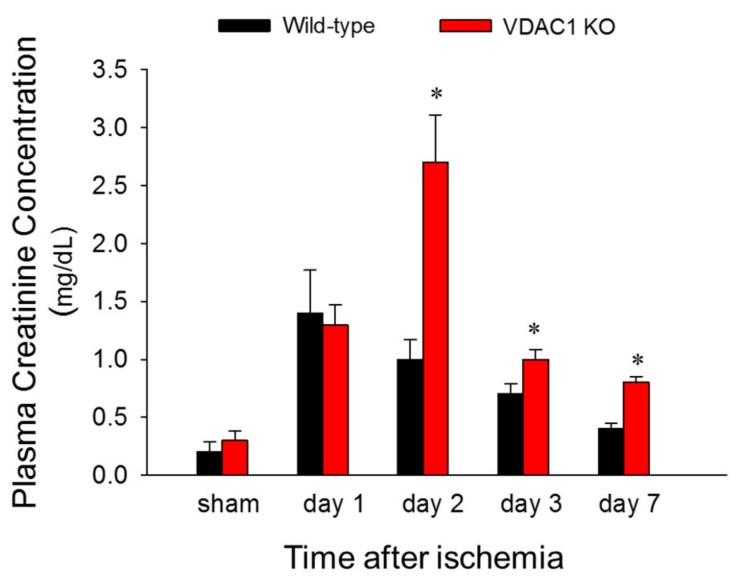
Deletion of VDAC1 delays recovery of renal function after ischemia. Renal function was evaluated using creatinine levels in mouse plasma collected from sham mice and at different time points during recovery after bilateral renal ischemia (35 min) in WT and VDAC1 KO mice. Results are the average ± S.E. of data obtained from 3–13 animals. * Values significantly different (*p* < 0.05) from respective sham controls.

To test if VDAC1 is involved in changes in renal function induced by ischemia, blood samples were taken at different times post reperfusion and the levels of plasma creatinine (a marker of renal function) were assessed. There was no difference in plasma creatinine levels between WT and VDAC1 KO sham mice. Creatinine levels increased 6-fold and 5-fold at 24 and 48 h after reperfusion, respectively, and recovered 7 days after ischemia in WT mice (Figure 2). VDAC1 deletion had no effect on the creatinine levels in sham mice (Figure 2). Creatinine levels increased 6-fold at 24 h after ischemia in VDAC1 KO mice and these increases were equivalent WT and VDAC KO animals (Figure 2). However, in contrast to WT mice, creatinine levels in VDAC1 KO mice continued to increase until 48 h post reperfusion when they were 2.7-fold higher than in WT mice and 10-fold higher than creatinine levels in VDAC1 KO sham controls (Figure 2). In contrast to WT mice, creatinine levels did not recover on day 7 after ischemia in VDAC1 KO mice, and were 3-fold higher than those prior to ischemia (Figure 2).

These data show that deletion of VDAC1 prolongs and exacerbates ischemia-induced decline in renal function and delays recovery of renal function after AKI.

### 3.3. VDAC1 Deletion Impedes the Recovery of Kidney Morphology After Ischemia

**Figure 3:**
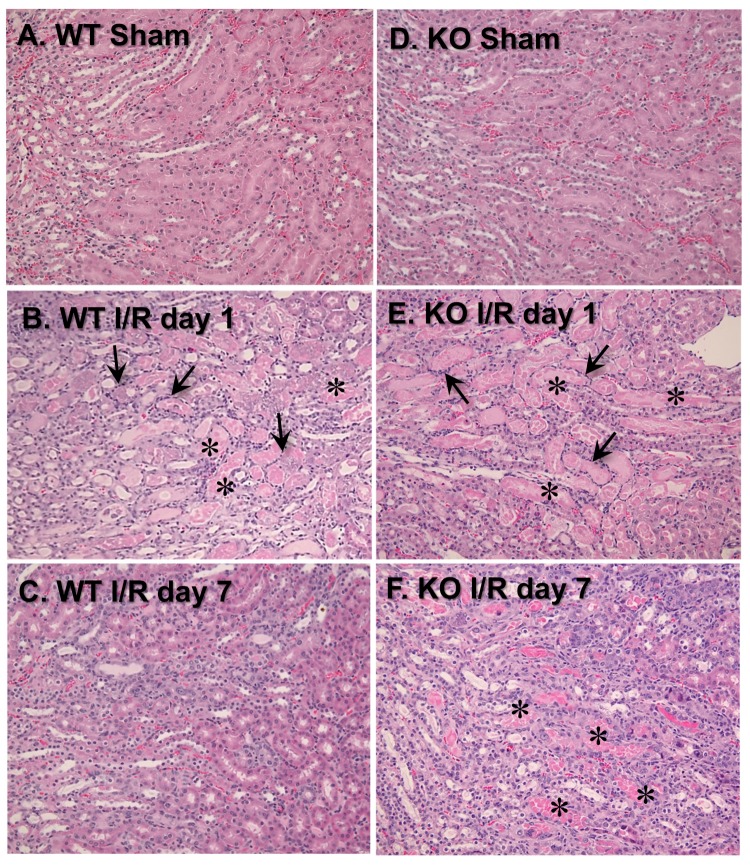
Deletion of VDAC1 exacerbates ischemia-induced damage to morphology of renal proximal tubules and impairs renal tissue regeneration after acute injury. On days 1 and 7 after ischemia, kidneys were harvested, immersed, fixed in 4% neutral-buffered formaldehyde, and embedded in paraffin. Thin (4–8 μm) sections were cut and stained with hematoxylin-eosin. Renal histology of the cortical-medullary junction was assessed in kidney sections from wild type (WT, **A**–**C**) and VDAC1-deficient (KO, **D**–**F**) mice. The images represent kidneys from sham-operated mice (**A**,**D**) and mice sacrificed on day 1 (**B**,**E**) and day 7 (**C**,**F**) after ischemia. * Necrotic tubules. The arrows mark inflammatory cell rings accumulated around necrotic tubules. The images were captured using Nikon Eclipse E800 microscope and Nikon 10× Plan Apo objective. Magnification: 122×. The sections are representative of kidneys from three WT and four KO sham-operated mice at each time point after ischemia (I/R) in different experiments.

To test the involvement of VDAC1 in morphological changes in ischemic kidneys, different histologic criteria were assessed on days 1 and 7 post reperfusion. Majority of morphological changes found in kidneys of WT mice at 24 h after ischemia occurred at the cortico-medullary junction (Figure 3A,B; Table 1). The changes involved severe tubular necrosis and loss of the brush border, tubular degeneration, and formation of copious tubular casts, significant damage to the distal nephron, and increased number of inflammatory cells (Figure 3A,B; Table 1). On day 7 post reperfusion, regeneration (relining) of damaged proximal tubules was nearly complete although some tubules did not have fully regenerated brush border and single tubular casts were scattered throughout the cortex in kidneys of WT mice (Figure 3C; Table 1).

VDAC1 deletion had no significant effect on the extent of morphologic damage to the proximal portion of the nephron and the number of inflammatory cells at 24 h after ischemia, but impaired recovery of proximal tubule morphology and tubular cast removal (Figure 3B,E; Table 1). In comparison with WT kidneys, regeneration of the proximal tubular part of the nephron in VDAC1-deficient kidneys was significantly reduced, necrotic foci and casts were apparent, and increased numbers of inflammatory cells were still present on day 7 post reperfusion (Figure 3C,F; Table 1).

Interestingly, the damage to the distal portion of the nephron was absent at 24 h and throughout the recovery period after ischemia in VDAC1-deficient kidneys (Figure 3B,E; Table 1). Thus, these data demonstrate that the absence of VDAC1 does not exacerbate morphological damage to the proximal tubular segment of the nephron, but impairs recovery of proximal tubule morphology after ischemia. These results suggest that VDAC1 plays an active role in the regeneration of the proximal segment of the nephron. In contrast, the absence of VDAC1 diminishes ischemic damage to the distal segment of the nephron.

### 3.4. The Absence of VDAC1 Promotes Interstitial Changes in Renal Tissue After Ischemia

**Figure 4:**
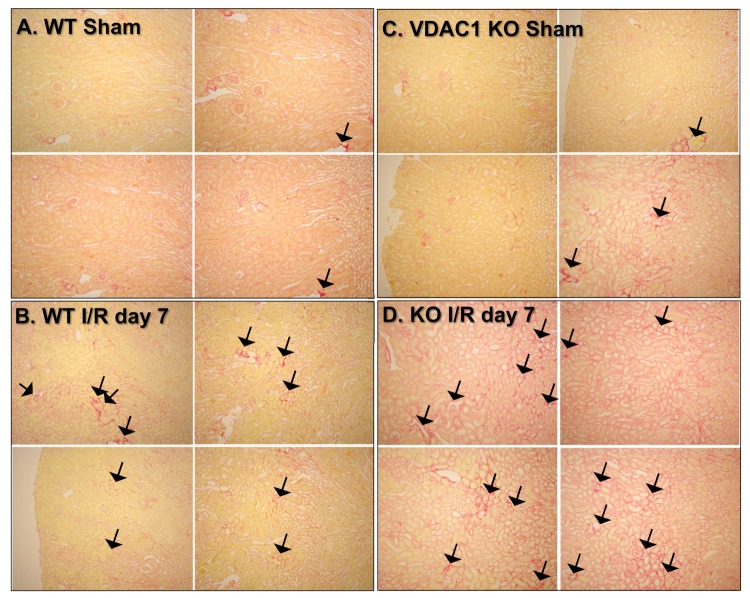
Deletion of VDAC1 exacerbates ischemia-induced interstitial collagen accumulation after acute injury. Kidneys were harvested before (sham) and on day 7 after ischemia (I/R) to assess collagen accumulation in the tissue. Formalin-fixed and paraffin-embedded 4–8-μm tissue sections were stained with picro-sirius red. Collagen staining in the renal interstitium was assessed by light microscopy using circularly polarized light. The images were captured using Nikon Eclipse E800 microscope using Nikon 10× Plan Apo objective. Magnification: 122×. (**A**) Representative kidney sections from three wild-type (WT) sham mice. (**B**) Representative kidney sections from four wild-type (WT) mice on day 7 after ischemia. (**C**) Representative kidney sections from three sham VDAC1-deficient (KO) mice. (**D**) Representative kidney sections from four VDAC1-deficient (KO) mice on day 7 after ischemia. The arrows mark sites with increased collagen accumulation.

Acute kidney injury is often followed by augmented accumulation of extracellular matrix proteins, which often results in interstitial fibrosis. Interstitial fibrosis has been implicated in the mechanisms of progression from AKI to the incident chronic kidney disease [36]. We hypothesized that impaired recovery of kidney morphology and function after AKI in VDAC1 KO mice might be associated with increased deposition and accumulation of extracellular matrix proteins in the kidney interstitium. Thus, we assessed the effect of VDAC1 deficiency on collagen accumulation in the renal interstitium after ischemia using picro-sirius red staining. In comparison with the sham kidneys, positive red staining representing collagen presence in the interstitium was slightly increased in several areas of WT kidneys on day 7 after ischemia (Figure 4A,B). VDAC1 deletion had no effect on collagen deposition in sham kidneys; however, it increased collagen accumulation in injured kidneys during the 7 day recovery period (Figure 4D). These results show that the absence of VDAC1 increases accumulation of extracellular matrix proteins in the injured kidney and suggest that the lack of VDAC1 exacerbates ischemia-induced kidney fibrosis.

### 3.5. Deletion of VDAC1 Reduces Survival After Renal Ischemia

**Figure 5:**
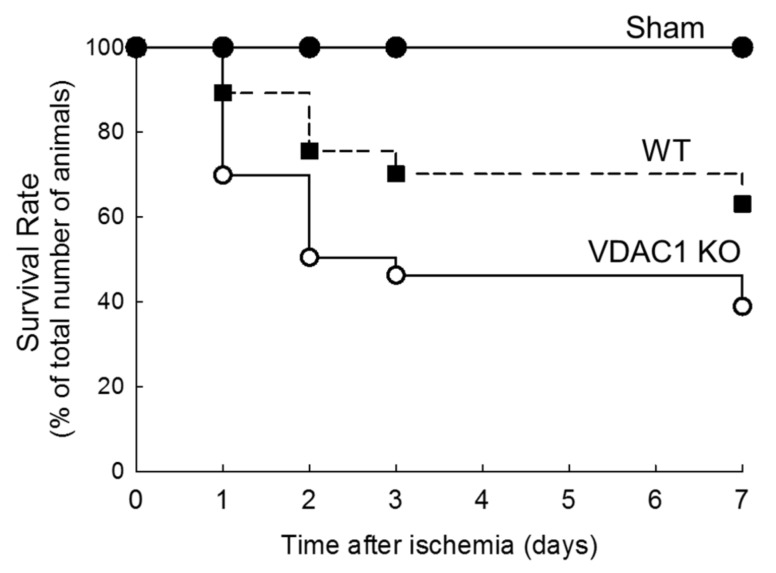
Deletion of VDAC1 decreases survival rate after ischemia-induced acute kidney injury. The comparison of survival of wild type (WT) and VDAC1-deficient (VDAC1 KO) mice subjected to bilateral ischemia (35 min) followed by reperfusion.

The survival rates were recorded to assess if deletion of VDAC1 and impaired recovery of kidney morphology and functions alter mice survival after ischemia. Figure 5 shows that the highest mortality rate in both WT and VDAC1 KO mice occurred within 48 h after ischemia. The animal loss within this critical period was 24% in WT and 50% in VDAC1 KO mice. On the seventh day after ischemia, mortality rate was 37% and 61% in WT and VDAC1 KO mice, respectively (Figure 5). No animals were lost from the sham-operated group. These results show that the deletion of VDAC1 increases (by 65%) mortality due to AKI and suggest that functional VDAC1 plays a critical role in survival after AKI (Figure 5).

### 3.6. VDAC1 Deficiency Abrogates Recovery of Mitochondrial Respiration After Ischemia

The recovery of kidney morphology and functions after injury is an energy-consuming process that requires adequate ATP supply by mitochondria. Mitochondrial function is disrupted by ischemia and has to recover prior to the return of renal functions to meet the demands for ATP in the regenerating kidney. VDAC1 facilitates the movement of crucial solutes, substrates, and products of oxidative phosphorylation between the cytoplasm and mitochondria. Therefore, we examined the importance of this channel for the recovery of oxidative phosphorylation in the renal cortical mitochondria after ischemia-induced AKI.

**Figure 6:**
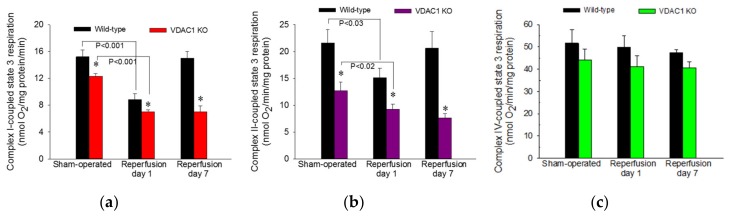
Deletion of VDAC1 decreases maximum mitochondrial respiration in non-injured renal cortical tissue and hinders recovery of respiration after ischemia. State 3 respiration coupled to the oxidation of electron donors to complex I (5 mM glutamate + 5 mM malate) (**a**), complex II (10 mM succinate + 0.1 μM rotenone) (**b**), and complex IV (2 mM ascorbic acid + 1 mM N,N,N’,N’-tetramethyl-p-phenylenediamine) (**c**) in renal cortical mitochondria isolated from sham-operated mice and mice on days 1 and 7 after bilateral renal ischemia (35 min) in wild type (WT) and VDAC1-deficient (VDAC1 KO) mice. Results are the average ± S.E. of data obtained from 4–11 animals. * Values in VDAC1-deficient renal cortical mitochondria significantly different (*p* < 0.05) from respective WT mitochondria.

ADP-stimulated state 3 respiration was used as a marker of the mitochondrial respiration associated with ADP phosphorylation and ATP synthesis in mitochondria isolated from renal cortical cells. State 3 respiration energized by glutamate and malate (substrates that generate NADH oxidized by complex I of the respiratory chain) was decreased to 58% of sham controls at 24 h after ischemia and recovered by day 7 after ischemia in WT mice (Figure 6a). In non-injured kidneys, deletion of VDAC1 decreased state 3 respiration to 81% of that in WT mice (Figure 6a). Ischemia in VDAC1 KO kidneys induced decreases in complex I-coupled state 3 respiration that were proportional to the decreases in WT kidneys (57% of sham controls at 24 h after reperfusion). However, in contrast to full recovery of respiration in WT kidneys, complex I-coupled state 3 respiration in VDAC1 KO renal cortical mitochondria did not recover and remained at the decreased level observed a day after ischemic injury (Figure 6a).

State 3 respiration energized by the oxidation of succinate though complex II of the electron transport chain decreased to 70% of controls at 24 h and recovered within 7 days after ischemia in WT kidneys (Figure 6b). Deletion of VDAC1 decreased complex II-coupled state 3 respiration in non-injured (sham) kidneys to 59% of that in WT sham mice. Ischemia produced further reduction in complex II-coupled state 3 respiration in VDAC1 KO animals. In contrast to full recovery of succinate-energized state 3 respiration in WT kidneys, state 3 respiration in VDAC1-deficient kidneys did not recover within 7 days post reperfusion and remained at the level observed at 24 h after ischemia (Figure 6b). Neither ischemia nor deletion of VDAC1 had any significant effects on state 3 respiration energized by electron donors to complex IV (Figure 6c).

These results demonstrate that VDAC1 deficiency decreases state 3 respiration in non-injured mitochondria and suggest that the access of substrates to mitochondrial matrix and the rate of electron flow through the respiratory chain are reduced in VDAC1 deficient kidneys. Moreover, the absence of VDAC1 blocks recovery of state 3 respiration after injury suggesting diminished capacity of VDAC1-deficient kidneys to produce ATP. Finally, these data show that VDAC1 is indispensable for efficient recovery of ADP-stimulated mitochondrial respiration following ischemia.

### 3.7. VDAC1 Deletion Hinders Recovery of Activities of Respiratory Complexes After Ischemia

To test whether decreases in state 3 respiration in VDAC1 KO mice are caused by a dysfunction of respiratory complexes, activities of complexes I, II, and III were assessed in renal mitochondria isolated from cortices of non-injured and ischemia-injured kidneys. The activity of complex IV was not measured because complex IV-coupled state 3 respiration was not altered in VDAC1-deficient kidneys.

**Figure 7:**
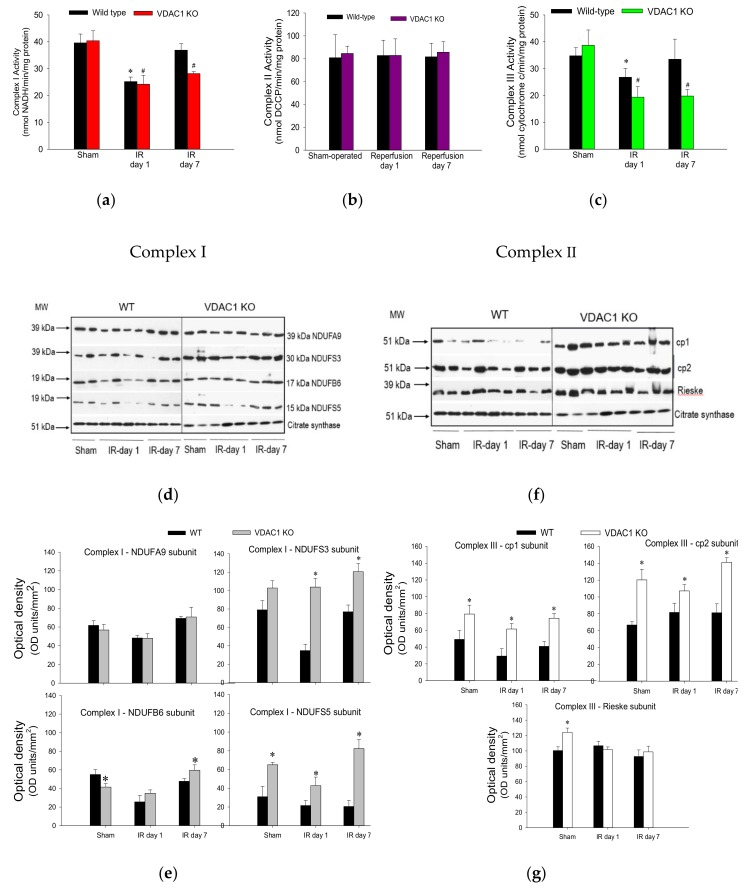
Deletion of VDAC1 impairs recovery of activities of complexes I and III of the electron transport chain in the renal cortex after ischemia-induced acute kidney injury. Activities of NADH:Ubiquinone Oxidoreductase (complex I) (**a**), Succinate:Ubiquinone Oxidoreductase (complex II) (**b**), and Ubiquinol:Cytochrome c Oxidoreductase (**c**) in mitochondria isolated from renal cortices of wild type (WT) and VDAC1-deficient (VDAC1 KO) mice on days 1 and 7 after bilateral renal ischemia (35 min). Protein levels (**d**) and densitometric analysis of immunoblots (**e**) of subunits NDUFA9, NDUFS3, NDUFB6, and NDUFS5 of NADH:ubiquinone oxidoreductase at different time points of recovery after bilateral renal ischemia (35 min) in wild type (WT) and VDAC1-deficient (VDAC1 KO) mice. Protein levels (**f**) and densitometric analysis of in immunoblots (**g**) of core protein 1 (cp1), core protein 2 (cp2), and Rieske subunit of ubiquinol:cytochrome c oxidoreductase at different time points of recovery after bilateral renal ischemia (35 min) in wild type (WT) and VDAC1-deficient (VDAC1 KO) mice. Results shown in (**a**–**c**) are the average ± S.E. of data obtained from five animals. * Values significantly different (*p* < 0.05) from respective WT sham-operated mice. # Values significantly different (*p* < 0.05) from respective VDAC1 KO sham-operated mice. The immunoblots (**d**,**f**) and their densitometric analysis (**e**,**g**) represent renal cortical mitochondria obtained from three sham-operated animals and three mice sacrificed on days 1 and 7 post reperfusion in three different experiments.

Ischemia followed by 24 h reperfusion decreased activity of complex I in WT kidneys to 73% of the sham WT controls (Figure 7a). This decrease was accompanied by a decline in protein levels of subunits NDUFA9 (39 kDa), NDUFS3 (30 kDa), NDUFB6 (17 kDa), and NDUFS5 (15 kDa) (Figure 7d,e). The activity of complex I recovered by day 7 of post reperfusion, which was associated with the recovery of protein levels of its four subunits (Figure 7a,d,e). Deletion of VDAC1 had no effect on the activity or protein levels of complex I in mitochondria isolated from non-injured kidneys (Figure 7a,d,e). The ischemia-induced decreases in complex I activity were similar in WT and VDAC1-deficient kidneys at 24 h post-reperfusion (Figure 7a). However, in contrast to the recovery of complex I activity after ischemia in WT kidneys, activity of this complex in VDAC1-deficient kidneys did not return and, on day 7 post reperfusion, it was 30% lower than that in respective sham controls (Figure 7a). Interestingly, protein levels of NDUFA9, NDUFS3, and NDUFB6 subunits of complex I were not altered by ischemia or reperfusion in VDAC1-deficient kidneys, which suggests that the progressive decline and the lack of recovery of the activity of complex I after ischemia were not due to reduced levels of complex I in mitochondria (Figure 7d,e).

Neither ischemia nor deletion of VDAC1 had any effect on the activity of complex II of the respiratory chain (Figure 7b). These data suggest that the declines in mitochondrial state 3 respiration caused by ischemia and the deficiency of VDAC1 are not caused by insufficient protein levels of complex II in the mitochondria.

The activity of complex III in renal cortical mitochondria of WT mice declined to 61% of sham controls at 24 h after ischemia and recovered on day 7 of post reperfusion period (Figure 7c). Deletion of VDAC1 had no effect on the activity of complex III in mitochondria isolated from non-injured kidneys but increased protein levels of subunits cp1, cp2, and Rieske iron-sulphur protein of complex III (Figure 7c,f,g). Ischemia decreased the activity of complex III in VDAC1-deficient kidneys to 40% of respective sham controls without having a substantial effect on the protein levels of the major subunits of this complex (Figure 7c,f,g). In addition, deletion of VDAC1 hindered recovery of complex III activity and, on day 7 post reperfusion, the activity of complex III was 30% lower than that in sham controls although protein levels of the major subunit of this complex were equivalent to those in sham kidneys and higher than in WT kidneys (Figure 7c).

These results show that the lack of VDAC1 has a differential effect on the activities of complexes of the electron transport chain in mitochondria of non-injured, injured, and regenerating kidneys. The deficiency of VDAC1 has no effect on the activities of complexes I and II, and increases mitochondrial levels and activity of complex III. Moreover, the absence of VDAC1 blocks recovery of activities of complexes I and III after ischemia without reducing their protein levels. These results suggest that maintaining the conductance of VDAC1 is critical for the recovery of normal functions of the electron transport chain and mitochondrial respiration after ischemia and that the return of activities of complexes I and III is dependent on the functional VDAC1 channel. Our data also show that the absence of VDAC1 leads to elevated levels of important subunits of complex I and complex III, but these increases do not lead to augmented activity of these two complexes.

### 3.8. Deficiency of VDAC1 Impairs Recovery of F_O_F_1_-ATPase After Ischemia

**Figure 8:**
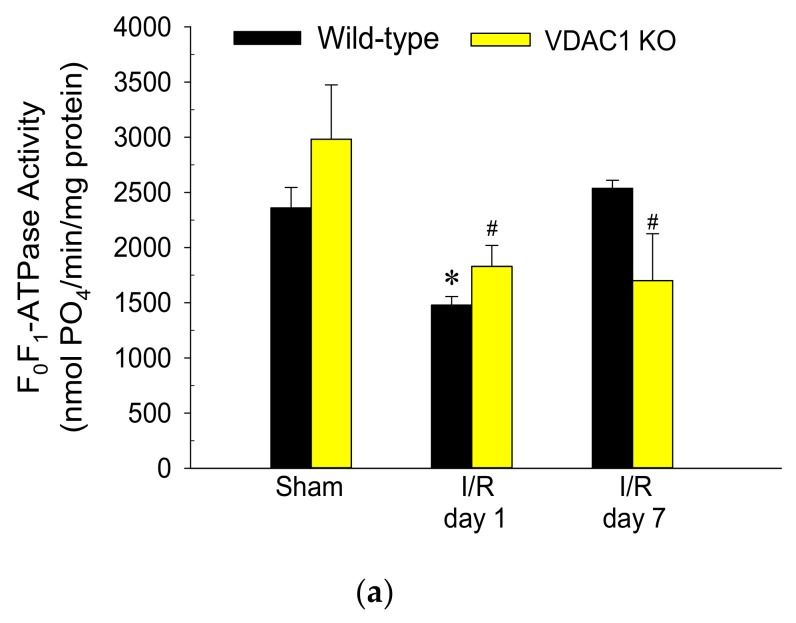

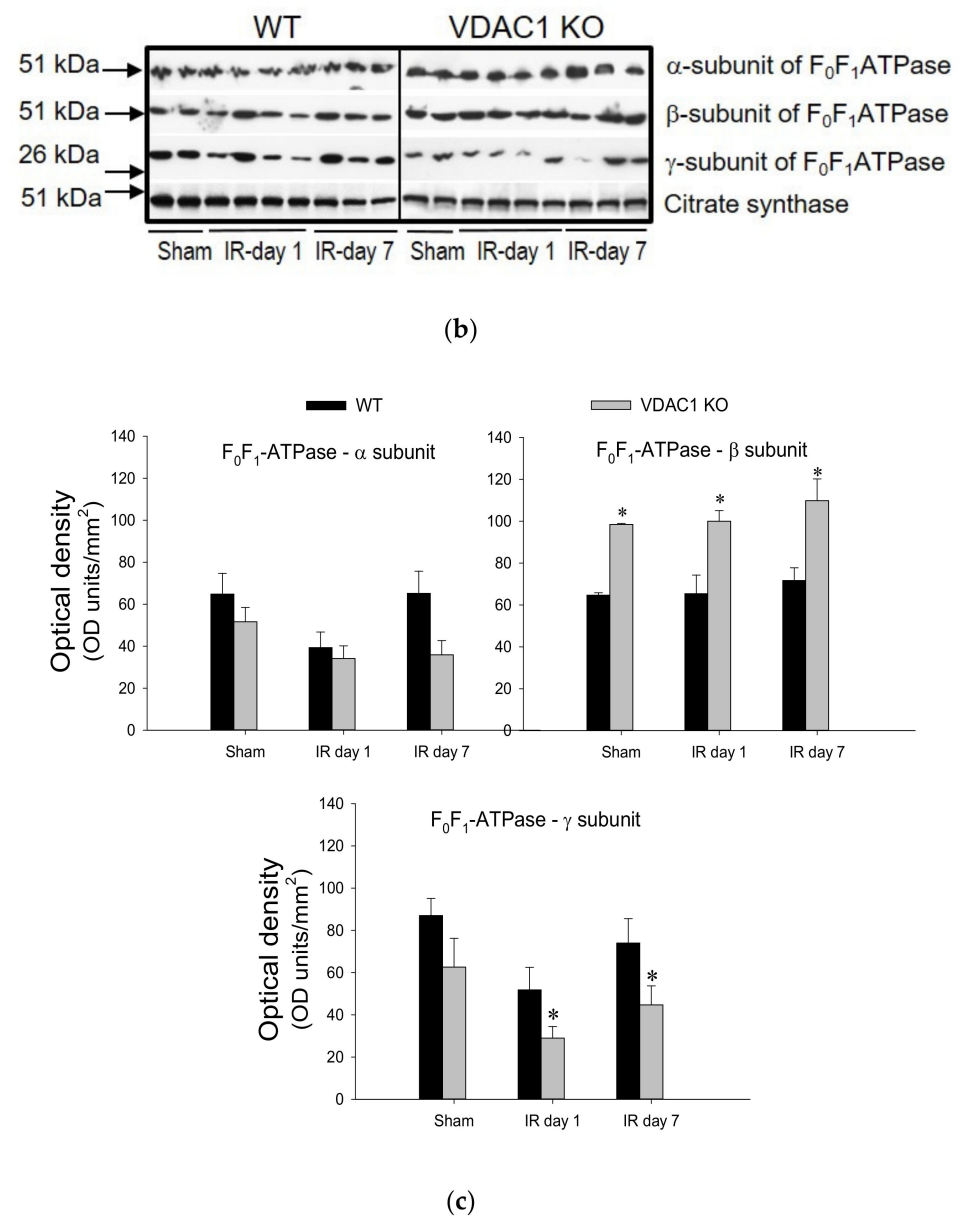
Deletion of VDAC1 blocks recovery of F_0_F_1_-ATPase activity in the renal cortex after ischemia-induced acute kidney injury (AKI). (**a**) Activity of F_0_F_1_-ATPase in mitochondria isolated from renal cortices of wild type (WT) and VDAC1-deficient (VDAC1 KO) mice on days 1 and 7 after bilateral renal ischemia (35 min). Results are the average ± S.E. of data obtained from 4–7 animals. * Values significantly different (*p* < 0.05) from respective WT sham-operated mice. # Values significantly different (*p* < 0.05) from respective VDAC1 KO sham-operated mice. (**b**) Protein levels of subunits α, β, and γ of the F_1_ (catalytic) domain of F_0_F_1_-ATPase during recovery after bilateral renal ischemia (35 min) in WT and VDAC1 KO mice. The blots show representative results from renal cortical mitochondria in three sham-operated mice, four mice sacrificed on day 1, and three mice sacrificed on day 7 post reperfusion in different experiments. (**c**) Densitometric analysis of immunoblots shown in (**b**). * Values significantly different (*p* < 0.05) from respective WT mice.

This study also tested if VDAC1 plays a role in the decreases of F_0_F_1_-ATPase activity that occurs in AKI and recovery of F_0_F_1_-ATPase after AKI. Activity of F_0_F_1_-ATPase in mitochondria isolated from renal cortices of WT mice decreased to 65% of sham controls at 24 h after ischemia, was associated with the decreases in protein level of the γ-subunit of the enzyme, and recovered within 7 days post reperfusion (Figure 8).

Deficiency of VDAC produced a 1.3-fold increase in F_0_F_1_-ATPase activity in non-injured kidneys and was accompanied by increased protein levels of the β (catalytic) subunit of the enzyme in renal cortical mitochondria (Figure 8). Ischemia induced a decline in F_0_F_1_-ATPase activity in VDAC1-deficient kidneys similar to that in WT kidneys (Figure 8). However, in contrast to the return of F_0_F_1_-ATPase activity after ischemia in WT kidneys, the F_0_F_1_-ATPase activity did not recover in VDAC1 KO kidneys and remained at the decreased level during the 7 days post reperfusion (Figure 8). Lack of recovery of F_0_F_1_-ATPase activity after ischemia in VDAC1-deficient kidneys was accompanied by decreased mitochondrial levels of its γ-subunit (the central shaft connecting the F_o_ and F_1_ subunits) in both non-injured and injured kidneys when compared to WT animals.

These data show that functional VDAC1 is essential for the full recovery of the catalytic activity of renal F_0_F_1_-ATPase after ischemia-induced AKI.

### 3.9. Deficiency of VDAC1 Reduces Renal Cortical ATP Content

**Figure 9:**
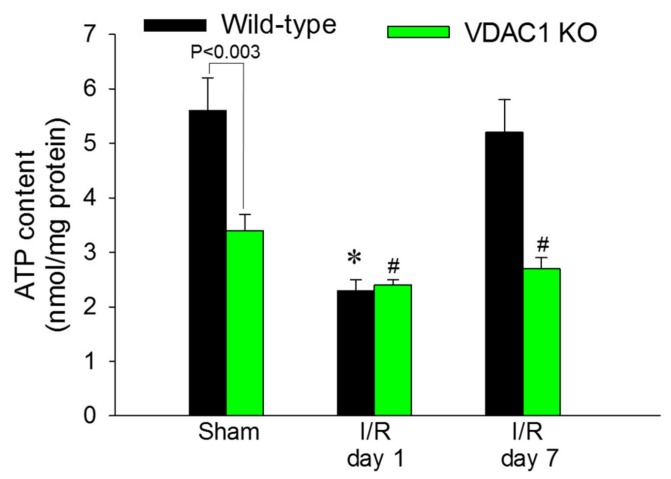
Deletion of VDAC1 blocks recovery of ATP content in renal cortex after ischemia-induced AKI. The content of ATP was assessed in liquid nitrogen snap-frozen renal cortical samples dissected immediately after euthanasia from kidneys of wild type (WT) and VDAC1-deficient (VDAC1 KO) mice on days 1 and 7 after bilateral renal ischemia (35 min). Results are the average ± S.E. of data obtained from eight WT and 11 VDAC1 KO mice. * Values significantly different (*p* < 0.05) from respective WT sham-operated mice. # Values significantly different (*p* < 0.05) from respective kidneys of VDAC1 KO sham-operated mice.

ATP content in renal cortex was assessed to test whether decreases in state 3 respiration and F_0_F_1_-ATPase activity in VDAC1-deficient kidneys result in reduced levels of tissue ATP content. The slices of renal cortex were dissected immediately after the heart function ceased and were instantaneously denatured in ice-cold acid to prevent ATP consumption and hydrolysis. Renal cortical ATP levels in WT mice declined to 41% of sham controls at 24 h after reperfusion and recovered by day 7 after ischemia (Figure 9).

VDAC1 deletion resulted in a 44% decrease in ATP content in cortices of non-injured kidneys in comparison with WT kidneys (Figure 9). Ischemia reduced ATP content in kidneys of WT and VDAC1 KO mice to the same level (2.4 ± 0.1 vs. 2.3 ± 0.2 nmol/mg protein in renal cortical tissue of VDAC1 KO and WT mice, respectively). However, deletion of VDAC1 blocked the recovery of renal ATP levels after ischemia and on day 7 post reperfusion, ATP content in VDAC1-deficient kidneys has not reached the levels found in sham controls (Figure 9). These data show that the presence of VDAC1 is crucial for maintaining ATP levels in the non-injured kidney. Furthermore, our data show that the lack of VDAC1 does not exacerbate ischemia-induced decreases in ATP, but blocks recovery of cellular ATP content after injury.

### 3.10. Lack of VDAC1 Is Associated with Mitochondrial Fission

**Figure 10:**
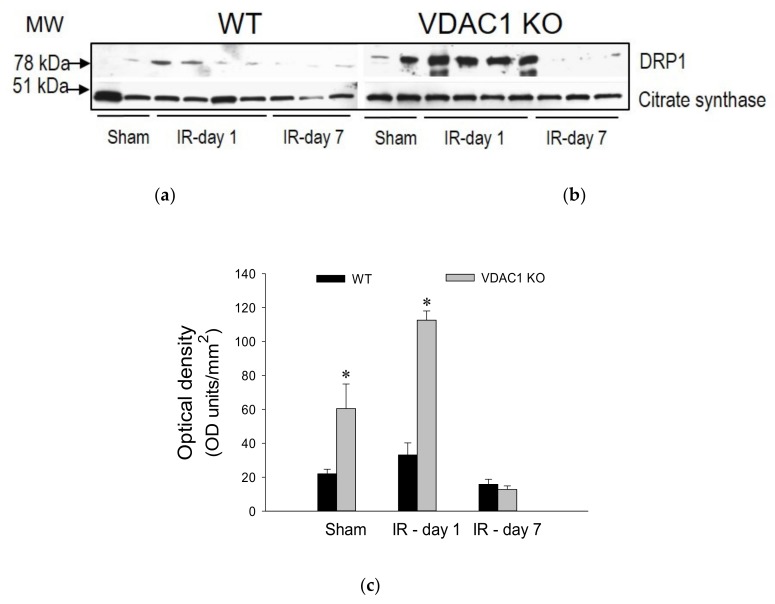
Deletion of VDAC1 induces mitochondrial fission in non-injured and ischemia-injured renal cortex. Protein levels of dynamin-related protein 1 (DRP1) on days 1 and 7 of recovery after bilateral renal ischemia (IR) in WT (**a**) and VDAC1 KO mice (**b**). (**c**) Densitometric analysis of the immunoblots shown in (**a**) and (**b**). The blots represent renal cortical mitochondria obtained from sham-operated animals and four mice sacrificed on day 1 (IR-day 1) and 3 mice sacrificed on day 7 (IR-day 7) post reperfusion in three different experiments. * Values significantly different (*p* < 0.05) from the mitochondria of respective WT mice.

Mitochondrial dysfunction is often associated with disruption of mitochondrial dynamics and increased fission (fragmentation) of this organelle. Drp1 is recruited to the mitochondria and binds to its corresponding receptors located on the mitochondrial outer membrane. Therefore, we used mitochondrial levels of DRP1 as a marker of the pool of fragmented mitochondria or mitochondria undergoing fission. No significant mitochondrial fission was present in non-injured kidneys of WT mice (Figure 10a). Mitochondrial fission was observed after ischemia in WT kidneys and the fragmentation of mitochondria returned to undetectable levels on day 7 after injury (Figure 10a).

In contrast, deficiency of VDAC1 was associated with increased levels of DRP1 in cortical mitochondria of non-injured kidneys, which suggested mitochondrial fragmentation in the absence of any injury (Figure 10b). Ischemia augmented mitochondrial levels of DRP1 (Figure 10b). These data show that ischemia induces association of DRP1 with renal cortical mitochondria suggesting alterations in mitochondrial dynamics and fragmentation. The lack of VDAC1 is sufficient to induce association of DRP1 with mitochondria in the absence of ischemic injury and promotes mitochondrial fission whereas ischemia augments this increase. These data suggest that VDAC1 plays a major role in the maintenance of mitochondrial dynamics in renal mitochondria.

## 4. Discussion

The VDAC1 channel is required for the movement of metabolites, including respiratory substrates, adenine nucleotides, Pi, and ions between the cytosol and mitochondria to maintain physiological functions of mitochondria [42,43]. Interestingly, changes in VDAC1 conductance do not affect creatine or phosphocreatine movements, which promote energy transfer through the phosphocreatine pathway [44]. In addition to regulating mitochondrial energy metabolism, VDAC1 plays a role in cell injury and death, both necrotic and apoptotic cell death [44,45,46]. Additionally, VDAC1 plays an important role in the release of mitochondrial reactive oxygen species that can initiate or exacerbate cell and organ injury [28]. Consequently, changes in VDAC1 mitochondrial levels, oligomerization, and conductance can attenuate or exacerbate injury. It has been shown that diabetic nephropathy is accompanied by diminished levels of VDAC1 in the kidney [27]; however, the role of individual VDAC isoforms in diabetic nephropathy or in mitochondrial dysfunction associated with acute kidney injury have not been assessed. Here, we demonstrate the presence of all three VDAC isoforms in the renal cortical cell mitochondria and examine whether VDAC1 plays a role in ischemia-induced dysfunction of renal mitochondria and subsequent acute injury caused by ischemic events in the kidney.

All three known VDAC isoforms are present in the renal cortical mitochondria and the functions of VDAC2 and VDAC3 may partially compensate for the lack of VDAC1 in non-injured kidneys to facilitate morphological and functional development of the kidney. However, in the absence of VDAC1, oxidative phosphorylation and tissue ATP levels are significantly reduced, which demonstrates that VDAC2 and/or VDAC3 do not fully substitute for the functions of VDAC1 in the non-injured kidney. Maximum (state 3, ADP-driven) mitochondrial respiration in non-injured kidneys of VDAC1 KO mice is reduced without any effect of VDAC1 deletion on the activities of complexes of the electron transport chain. A limited movement of metabolites and substrates/products of oxidative phosphorylation across the outer mitochondrial membrane in the absence of VDAC1 could explain this outcome. The transport of these solutes and ions across the outer mitochondrial membrane must occur through another gateway(s) and VDAC2 and/or VDAC3 could serve as these channels in non-injured kidney. However, this transport is reduced due to the absence of VDAC1 and is sufficient to support respiration and ATP synthesis, but at levels reduced in comparison with those in WT kidneys.

Cellular regeneration and recovery of cell functions after injury requires increased supply of ATP to support DNA repair, recycling of damaged macromolecules and organelles, repair of cellular membranes, and synthesis of new proteins and other macromolecules. Our data show that VDAC1 is a major protein necessary for the recovery of mitochondrial functions, ATP levels, and kidney morphology and functions after injury. Deletion of VDAC1 blocks recovery of ATP levels after ischemic injury, morphological regeneration of proximal tubules, and the return of kidney functions. In contrast, deletion of VDAC1 appears to prevent distal tubule damage. Distal tubules have higher expression of glycolytic enzymes than proximal tubules and are less dependent on mitochondria to produce ATP [46]. Therefore, glycolysis can compensate for reduced oxidative phosphorylation when mitochondrial functions are compromised by ischemia. The absence of VDAC1 in proximal tubules does not exacerbate ischemia-induced decreases in state 3 respiration and the activities of complexes I and III, the two major targets of ischemia in the electron transport chain. However, deletion of VDAC1 blocks recovery of activities of these complexes following ischemia. This outcome was unexpected as protein levels of the major subunits of complex I and complex III in VDAC1-deficient injured kidneys were higher than in WT kidneys and the decreases in activities of these complexes could not be attributed to increased degradation or decreased synthesis of subunits of these two complexes. However, a substantial pool of these proteins was not functional after ischemia, which suggested that the subunits of complexes I and III were present, but their assembly was disrupted and could not be repaired in the absence of VDAC1. In contrast, the functions of complexes II and IV were unaffected by ischemia and lack of VDAC1. These data suggested that the hindered repair of complexes I and III, which have the major contribution to generation of the proton-motive force in mitochondria, blocked recovery of ATP production and content in injured kidneys lacking VDAC1. These results underscore the fact that VDAC1 is an important gateway for the entry of substrates and products of oxidative phosphorylation in injured proximal tubules and that the alternate channels do not allow for sufficient movement of these substrates/products during increased demand for energy in regenerating kidney. The lack of VDAC1 hinders recovery of ADP-driven respiration, ATP levels, morphology of the proximal tubules, and kidney functions after ischemic injury. These data support the conclusion that VDAC1 is an important VDAC isoform in renal cortical mitochondria and that it plays a major role in kidney recovery after ischemia-induced AKI.

Previously, we have shown that mitochondrial fragmentation (fission) in renal proximal tubular cells leads to reduced state 3 respiration, decreased activity of complexes I and III of the electron transport chain, hyperpolarization of the mitochondrial membrane, and reduced cellular ATP content [41,47]. Hence, we tested whether the absence of VDAC1 stimulates mitochondrial fission in non-injured and ischemia-injured mitochondria using the association of DRP1 with the mitochondria as a marker. DRP1 is an essential protein that mediates the final stage of mitochondrial fission by binding tightly to its specific receptors localized on the outer mitochondrial membrane. Wrapping of bound DRP1 molecules around the mitochondria leads to their fragmentation [48]. DRP1 was absent from the mitochondria isolated from cortices of non-injured WT kidneys. In WT kidneys, ischemia disrupted normal mitochondrial dynamics and slightly increased association of DRP1 with the mitochondria indicating mitochondrial fission. These changes were associated with disruption of respiration and integrity of the electron transport chain, and decreased ATP content in the renal cortex. Recovery of mitochondrial function after ischemia in WT mice was accompanied by decreased association with DRP1, which suggested reduced mitochondrial fission. On day 7 post reperfusion, mitochondrial levels of DRP1 were almost undetectable by immunoblotting and were accompanied by the recovery of respiration, activities of complex I, complex III, and F_0_F_1_-ATPase, and tissue ATP content.

In contrast, non-injured mitochondria lacking VDAC1 exhibited significant levels of DRP1 that were higher than the levels of DRP1 in injured WT mitochondria. It is noteworthy that the decreases in state 3 respiration and ATP content coincided with mitochondrial fission in non-injured kidneys of VDAC1 KO mice. Ischemia exacerbated increases in mitochondrial levels of DRP1 in VDAC1 KO kidneys suggesting increased mitochondrial fission in comparison with WT kidneys. The disruption of mitochondrial dynamics in VDAC1-deficient kidneys was accompanied by the lack of recovery of state 3 respiration, activities of complex I, complex III, and F_0_F_1_-ATPase, and ATP levels after ischemia. These changes occurred despite increased protein levels of complex III subunits and unchanged levels of subunits of complex I. This suggests that mitochondrial fission and dysfunction are induced by the deficiency of VDAC1 and not by decreased protein levels of complexes of the electron transport chain and subunits of the catalytic domain of ATP synthase (F_0_F_1_-ATPase). Increases in protein levels of complex III and the catalytic (β) subunit of F_0_F_1_-ATPase in mitochondria lacking VDAC1 may be a compensatory response to decreased activity of these enzymatic complexes and reduced oxidative phosphorylation or due to decreased degradation of these proteins. Overall, these results show that VDAC1 plays an important role in the regulation of mitochondrial dynamics and that the deficiency of this channel induces mitochondrial fission and reductions in functions in non-injured kidneys, and prevents recovery of mitochondrial functions and normal mitochondrial dynamics after ischemic injury.

In summary, our results show that ischemia decreases protein levels of VDAC1 in the renal cortical mitochondria and that VDAC1 levels return during the recovery period in WT mice. These decreases coincide with reduced mitochondrial and renal functions and ATP levels, increases in mitochondrial fission, morphological damage to the proximal segment of the nephron, and interstitial accumulation of collagen. The return of protein levels of VDAC1 after ischemia is accompanied by recovery of mitochondrial dynamics and functions, morphological regeneration of the kidney, and the return of normal kidney functions. Deletion of VDAC1 results in reduced mitochondrial respiration and ATP levels and increased mitochondrial fission in non-injured kidneys without any apparent effect on renal morphology and functions. However, the absence of VDAC1 in injured kidneys hinders recovery of mitochondrial functions and dynamics, impedes regeneration of renal morphology and return of kidney functions, and increases deposition of collagen in renal interstitium after ischemia. Our results also suggest that in the absence of VDAC1, the other two isoforms i.e., VDAC2 and/or VDAC3, play a compensatory role in the kidney physiology and pathophysiology.

## 5. Conclusions

We conclude that VDAC1 is a major VDAC isoform that supports recovery of mitochondrial functions, dynamics, and remodeling after renal ischemia. Not only is VDAC1 important for maintaining mitochondrial energy metabolism and dynamics in non-injured kidney, but it plays a major role in the full recovery of these functions, restoration of kidney morphology and functions, and survival after ischemia-induced AKI. We also conclude that VDAC1 plays an important role in morphological regeneration of the proximal tubule segment of the nephron and the absence of this channel hinders recovery of the proximal tubules and impedes the return of kidney functions after ischemia. Lastly, our results suggest that VDAC1 reduces progression of renal fibrosis after acute kidney injury.

## Figures and Tables

**Table 1:** Changes in kidney morphology in wild-type (WT) and VDAC1-deficient (KO) mice during recovery after bilateral renal ischemia. Morphological damage to the kidney after ischemia/reperfusion (I/R) was evaluated by light microscopy using fixed kidney sections embedded in paraffin, processed for light microscopy, and stained with hematoxylin-eosin. The following histologic criteria were used to assess morphological damage to the kidney: tubular necrosis, brush border loss, tubular cast formation, degeneration, distal nephron damage, inflammation (infiltration of the inflammatory cells), and regeneration. These parameters were evaluated on a scale of 0–4: not present (0), mild (1), moderate (2), severe (3), and very severe (4). Results are presented as average ± S.E. of data obtained from kidney sections from wild-type (WT, *n* = 3) and VDAC1-deficient mice (KO, *n* = 4). * Values significantly different (*p* < 0.05) from WT.

Days After I/R	Tubular Necrosis		Brush Border Loss		Cast Formation		Degeneration	
	WT	KO	WT	KO	WT	KO	WT	KO
Sham	0.0 ± 0.0	0.0 ± 0.0	0.0 ± 0.0	0.0 ± 0.0	0.5 ± 0.3	0.0 ± 0.0	0.0 ± 0.0	0.0 ± 0.0
Day 1	4.0 ± 0.01	3.6 ± 0.2	2.7 ± 0.6	2.3 ± 0.4	2.7 ± 0.6	3.5 ± 0.4	1.7 ± 0.5	2.8 ± 0.4
Day 7	0.3 ± 0.2	2.8 ± 0.4 *	1.0 ± 0.0	2.3 ± 0.6 *	1.0 ± 0.0	3.3 ± 0.9 *	0.0 ± 0.0	0.8 ± 0.9

	**Distal Nephron Damage**		**Inflammation**		**Regeneration**			
	WT	KO	WT	KO	WT	KO		
Sham	0.0 ± 0.0	0.0 ± 0.0	0.0 ± 0.0	0.0 ± 0.0	0.0 ± 0.0	0.0 ± 0.0		
Day 1	3.3 ± 0.5	0.1 ± 0.1 *	3.0 ± 0.0	3.0 ± 0.1	0.0 ± 0.0	0.0 ± 0.0		
Day 7	0.0 ± 0.0	0.5 ± 0.3	0.3 ± 0.3	1.3 ± 0.3 *	3.7 ± 0.3	2.0 ± 0.7 *		
